# Architecture of the Synaptophysin/Synaptobrevin Complex: Structural Evidence for an Entropic Clustering Function at the Synapse

**DOI:** 10.1038/srep13659

**Published:** 2015-09-03

**Authors:** Daniel J. Adams, Christopher P. Arthur, Michael H. B. Stowell

**Affiliations:** 1 Department of MCD Biology, University of Colorado, Boulder, CO 80309; 2 Department of Mechanical Engineering, University of Colorado, Boulder, CO 80309.

## Abstract

We have purified the mammalian synaptophysin/synaptobrevin (SYP/VAMP2) complex to homogeneity in the presence of cholesterol and determined the 3D EM structure by single particle reconstruction. The structure reveals that SYP and VAMP2 assemble into a hexameric ring wherein 6 SYP molecules bind 6 VAMP2 dimers. Using the EM map as a constraint, a three dimensional atomic model was built and refined using known atomic structures and homology modeling. The overall architecture of the model suggests a simple mechanism to ensure cooperativity of synaptic vesicle fusion by organizing multiple VAMP2 molecules such that they are directionally oriented towards the target membrane. This is the first three dimensional architectural data for the SYP/VAMP2 complex and provides a structural foundation for understanding the role of this complex in synaptic transmission.

Synaptic vesicles (SV) are the organelles that traffic neurotransmitter to the synaptic cleft and propagate signals between neurons upon fusion with the plasma membrane. The SV lifecycle is complex and carefully regulated from endocytosis, maturation and neurotransmitter loading to docking and release. The fusion event is mediated by the interaction of the v-SNARE synaptobrevin2 (VAMP2) with the t-SNAREs syntaxin-1/SNAP-25^1^. Disruption at any point in the SV cycle can result in dysfunction leading to a myriad of neurological and neurodegenerative disorders^2^,^3^,^4^. Synaptophysin (SYP) was one of the first synaptic proteins identified more than 40 years ago^5^,^6^ yet its biochemical function has remained elusive and ascribing a clear role in the synaptic vesicle cycle has been absent. It has however been demonstrated that knockout animals developmentally compensate for the loss of SYP through the expression of several paralogs^7^,^8^ and modest effects on working memory and SV endocytosis have been reported^9^,^10^, suggesting that SYP may play a role in the synaptic vesicle cycle. More recently, the use of an optogenetic method using a miniSOG fusion of both SYP and VAMP2 demonstrated that light inactivation of SYP resulted in a subsequent greater inhibition of synaptic release than light inactivation of VAMP2 in cultured hippocampal neurons. However, the specificity of this method has not been fully demonstrated and inactivation of other synaptic proteins in proximity to the miniSOG could have occurred^11^. SYP is a 4-pass integral membrane protein^12^ that forms a hexameric channel-like structure^13^. SYP comprises ~10% of the SV proteome by weight^14^ and it has been proposed that SYP forms a calcium sensitive channel^15^,^16^. SYP is ubiquitously expressed in synapses throughout the mammalian brain^17^ and is conserved from humans to nematodes^18^ yet SYP −/− mice lack an obvious phenotype^10^,^19^. SYP is a member of the physin protein family (Fig. 1) which consists of SYP, synaptoporin (SYNPR), pantophysin (SYPL1), mitsugumin (SYPL2), and synaptogyrins 1–4 (SNG1–4)^7^,^20^ and developmental compensation by the paralogs could explain the lack of a clear phenotype in the SYP −/− mice. Although the biological function of SYP is unclear, SYP is known to bind cholesterol^21^ and VAMP2 in SVs^22^, yet the importance of these interactions has been poorly understood. Recent evidence has suggested that SYP is involved in trafficking VAMP2 back into SVs during endocytosis^9^,^23^ and proper trafficking of VAMP2 from the plasma membrane to synaptic vesicles is the primary function of SYP. Here we report the first structural information on the native mammalian SYP/VAMP2 complex as derived from single particle EM and we have used this data to construct an atomic model of the SYP/VAMP2 complex.

## Results

### VAMP2 and SYP form a cholesterol dependent 2:1 complex

**Figure 2:**
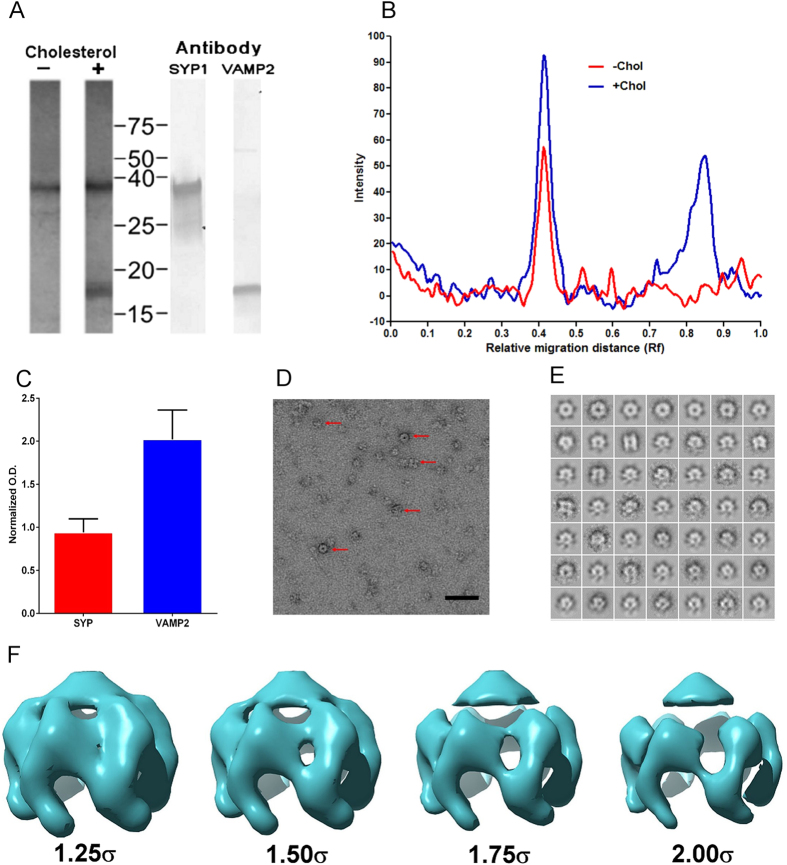
SYP/VAMP purification, stoichiometry and hexameric structure. (**A**) Native SYP and SYP/VAMP2 complex was purified from calf brain and analyzed by SDS-PAGE, silver stain and western blot. (**B**) Densitometry traces of protein bands from purification without cholesterol (red) or with cholesterol (blue). Purification with cholesterol showed a 1.37:1 mass ratio of SYP:VAMP2 consistent with a 6:12 stoichiometry which would be expected to show a 1.35:1 ratio based on total number of amino acids for each protein. (**C**) Normalized O.D. with standard deviations according to amino acid content and molecular weight from densitometry (**D**) Representative raw micrograph of negative stained protein with 5 sample particles indicated with red arrows. Scale bar = 50 nm. (**E**) Class averages used in the reconstruction. (**F**) Final EM map of the SYP/VAMP2 complex contoured at multiple levels (1.25, 1.50, 1.75 and 2.00 sigma) to highlight the presence of extra density presumed to correspond to VAMP2 in the complex reconstruction.

Purified SYP exists as a homo-hexamer resembling a channel pore^13^,^15^ and VAMP2 has been reported to exist as a dimer^24^,^25^ but it is not known if VAMP2 bound to SYP also exists as a dimer or what is the stoichiometry of the complex. We isolated native complexes from bovine brain to high purity by maintaining a high cholesterol environment during the purification. The complex had an apparent molecular weight of 400 kD based upon standards used to calibrate the sizing column. The stoichiometry of the isolated complex was 2:1 or 6 VAMP2 dimers bound to a single SYP hexamer as determined by a combination of size exclusion chromatography, SDS-PAGE, and western blot analysis of the purified complex (Fig. 2A–C).

### The SYP/VAMP2 Complex is Hexameric with 6 SYP and 6 VAMP2 dimers

**Figure 3:**
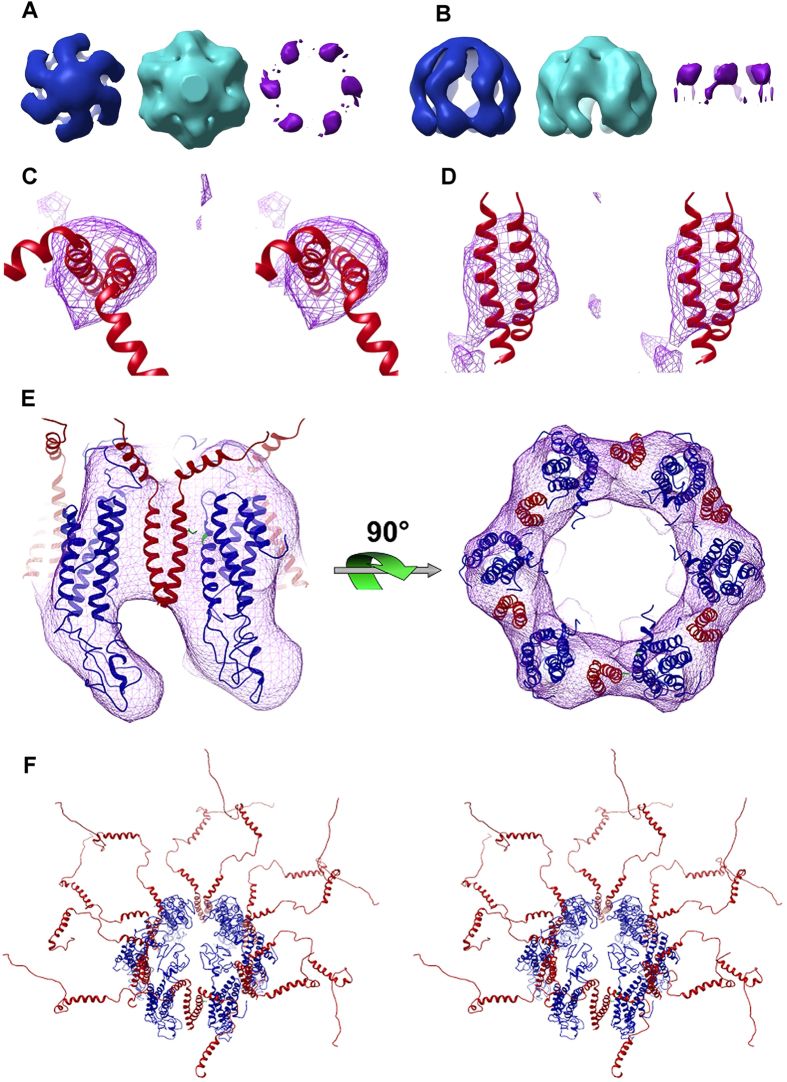
Synaptophysin clusters 6 VAMP2 dimers. View from cytoplasmic face (**A**) and view in the membrane plane (**B**) of EM density map of native SYP (blue)^13^ the SYP/VAMP2 complex (cyan) and the difference density attributed to the VAMP2 dimers (purple). Stereo view image from the cytoplasmic face (**C**) and view in the membrane plane (**D**) of the modeled VAMP2 dimer (red ribbons, PDB 2KOG) in the difference density map (purple wire mesh). (**E**) Atomic model of SYP (blue ribbon) and VAMP2 (red ribbon) docked into EM density map (wire mesh) at the determined 6:12 stoichiometry (**F**). Cross-eyed stereo image of the complete SYP_6_/VAMP2_12_ complex derived from fitting the EM map to the VAMP2 NMR structure (PDB 2KOG) and the refined SYP model produced in this work.

The SYPV/AMP2 complex isolated in high cholesterol was prepared for structural analysis by negative stain electron microscopy (Fig. 2D). We selected a total of 1432 particles for single particle reconstruction that allowed us to generate a final 3D density map (Figs 2E,F and 3). Figure 2F shows the final reconstruction contoured at several levels demonstrating the presence of extra density within the spokes of the SYP complex. In addition, difference map calculations further demonstrate the presence of additional density which approximates the dimeric transmembrane helices of VAMP2 located within the spokes of SYP (Fig. 3A–D). The difference density encompasses a total volume of 61,000 Å^3^ for the hexamer and 10,166 Å^3^ for a VAMP2 dimer. This is in good agreement with the expected volume of the VAMP2 transmembrane helix dimer of 12,000 Å^3^ calculated from the known NMR structure which shows multiple conformations for the extra-membrane domains of VAMP2 which are expected to be unobservable using single particle EM reconstruction. Bovine SYP and VAMP2 both share very high identity with the orthologous human proteins (94.6% and 99.1% respectively) so we expect that the structure at this resolution is general for the complex found in all mammals if not all vertebrates. The SYP/VAMP2 complex shows 6-fold symmetry that resembles the previously described SYP structure^13^ with six spokes radiating from a central hub. The outer diameter of the complex is 7.0 nm and the height measures roughly the same dimension. When compared with the previous SYP structure^13^ (Fig. 3) two important differences become evident. First, from the top (cytoplasmic face) there is new density between each of the six spokes of SYP which we attribute to the presence of VAMP2 dimer. Second, the density corresponding to the four-helix bundle of the SYP TMDs is slightly tilted in the complex relative to the structure of SYP alone, indicating a slight structural rearrangement presumably caused by binding of VAMP2 and/or cholesterol. This observed conformational change should be considered suggestive and not definitive because of the limited resolution of the current structure.

### Atomic model of the SYP/VAMP2 complex

Based on hydrophobicity alignment to the gap junction protein connexin, which is a structural homolog of the SYP hexamer, we were able to thread the SYP backbone onto the CX26 structure^26^, we then added appropriate rotamer sidechains and fit the model into our EM density map. Next, we docked the NMR structure of VAMP2^27^ into our model with the transmembrane helices of the dimer inserted between the SYP molecules to account for the additional density observed in our EM reconstruction. The SNARE domain of VAMP2 is expected to be very flexible and unobservable by single particle EM and accordingly was modeled outside of the density map. The resulting structural model of the full complex (Fig. 3E,F) ideally presents up to 6 VAMP2 dimers for SNARE formation and vesicle fusion. We believe that the overall architecture of our model accurately represents the complex found on native synaptic vesicles however the atomic details must be considered speculative given the low resolution of our structure. Nonetheless, certain predictions regarding the interaction of SYP and VAMP2 can be formulated. In particular, the G217R clinical mutation in SYP that is associated with X-Linked Intellectual Disability^28^ is found on the 4th transmembrane helix of SYP and appears at a predicted interface with the VAMP2 transmembrane helix. G217 of SYP is conserved in all vertebrates, and is also conserved in the paralogs SYPL1, SYPL2, SYNPR and SNG1. Furthermore, this position represents a predicted transmembrane pocket on SYP that may accommodate the transmembrane side chain of VAMP2 I98. Our structural model predicts that replacement of glycine with a relatively bulky and charged arginine at position 217 would attenuate the ability of SYP to bind with VAMP2. Recently, it was shown that introduction of the G217R mutation in SYP induces VAMP2 retrieval defects^29^ which may contribute to the developmental and cognitive impairments observed in patients with this mutation and is in concordance with our structural model.

## Discussion

**Figure 4:**
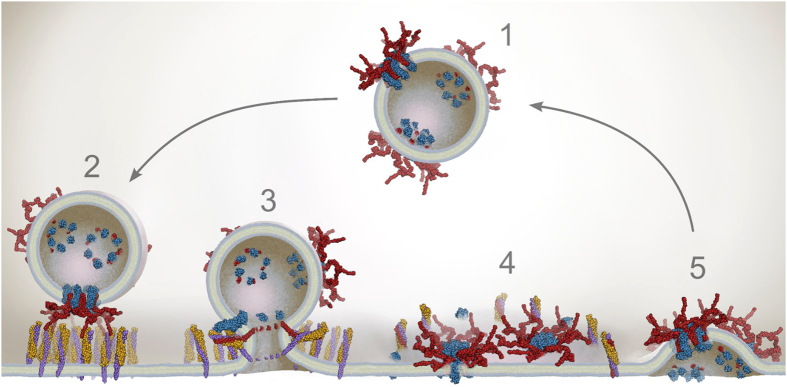
Proposed model of synaptophysin as an entropic catalyst. (**1**) Mature SVs contain 5 or 6 complete SYP/VAMP2 complexes. (**2**) SVs are trafficked to the active zone where accessory proteins mediate docking of clustered VAMP2 (red) with ordered arrays of the t-SNARE complex of syntaxin-1 (purple) and SNAP-25 (gold). (**3**) Calcium influx induces SNARE zippering as SYP (blue) dissociates from VAMP2, the fusion pore opens and neurotransmitter is released. (**4**) The post-fusion SNARE assemblies and disassociated SYP molecules ring the membrane patch contributed by the SV, which contains the intact SYP/VAMP2 complexes not involved in the fusion reaction. (**5**) NSF disassembles the SNARE bundles and t-SNAREs return to the active zone. SV components are loaded into clathrin coated pits where the SYP/VAMP2 complex reforms. Endocytosis recovers the SVs for subsequent loading and fusion.

The SYP/VAMP2 structure described here suggests a new model for life cycle of the synaptic v-SNARE VAMP2 (Fig. 4). Unlike other SNARE-mediated fusion events, SV release requires exquisitely tight temporal coupling of fusion with Ca^++^ influx. We propose that this event features additional catalytic mechanisms not found in general fusion pathways. Without pre-ordering SNAREs in the vesicle, exocytosis still requires multiple trans-SNARE contacts, but these can only be made at significant entropic cost that would impede physiologic fusion kinetics. This entropic cost is paid in electrostatic and hydrophobic forces upon binding to SYP early in the vesicle cycle to from the SYP/VAMP2 complex. This seemingly benign assembly realizes its potential by allowing a ring of 12 SNAREs to simultaneously dock at the membrane providing the cooperative and rapid opening of a fusion pore upon Ca^++^ influx. Based upon this proposed activity, SYP can be thought of as an entropic catalyst of neurotransmitter release that clusters multiple v-SNARES for rapid fusion. Assembly of the pre-fusion pore appears to be rate-limiting, consistent with evidence that structured arrays of t-SNARE proteins, which may mirror the structure presented here of the pre-fusion v-SNARE complex, serve as the docking sites of vesicles^30^,^31^,^32^. The SYP/VAMP2 interaction is labile relative to the bundled SNARE trimer and these are known to be mutually exclusive molecular contexts for VAMP2^33^. According to our model, this is essential to allow fusion when VAMP2 interacts with the t-SNARES and the SYP/VAMP2 binding is just strong enough to hold VAMP2 together until it docks with the t-SNAREs and accessory proteins. Recently it was reported that SV fusion with reconstituted membranes displayed comparable kinetics to synthetic liposomes^34^, however the conditions used in this experiment were not consistent with maintenance of the SYP/VAMP2 interaction due to freeze thawing of the SVs which is well known to disrupt the SYP/VAMP2 complex^33^. We predict that vesicles with natively reconstituted pre-fusion v-SNARE complexes of SYP/VAMP2 would show enhanced fusion kinetics in *in vitro* assays. To date this has not been tested because of the labile nature of the SYP/VMP2 complex. Evidence for any activity of SYP has remained elusive because multiple functional homologs can substitute for SYP in knockout animals and experimental conditions suitable for observing these effects are not readily available. Although reconstituted systems have allowed the observation of fusion mediated by single SNARE binding events^35^, it has recently been demonstrated that biologically relevant SV fusion rates require at least 6 SNARE interactions^36^,^37^,^38^. The contact patch of a fusing 50 nm diameter vesicle with the plasma membrane is roughly 180 square nm^39^,^40^ which, at ~60 copies per vesicle^1^,^14^, yields less than a single VAMP2 dimer occupying that area. While SNAREs extend beyond the surface of the vesicle by approximately 12 nm, which in principle could augment this contact patch area, this alone cannot generate coordinated binding of the required 6 SNARE pairs. Synaptophysin is found at ~30 copies per SV^14^ which combined with our results and previous studies^22^, suggests that the SYP/VAMP2 is the predominant form found on the synaptic vesicle. From this, we predict that on average five or six SYP/VAMP2 complexes stud the surface of every readily releasable SV. By pre-clustering VAMP2 into a stable complex, SYP not only allows for presentation of the requisite number of SNAREs, but also makes the individual trans-SNARE binding interactions cooperative. This physical model implies that the fusion pore would open at the center of the SYP hexamer and the inner diameter of our pore structure (~3.0 nm) and previous measurements of the large conductance (415 pS) of the SYP pore^41^ are consistent with direct measurements of the synaptic vesicle fusion pore^42^ of ~2.3 nm and a conductance in excess of 375 pS. These results support the hypothesis that the SYP/VAMP2 complex represents the physiologic pre-fusion state of the v-SNARE which, together with the docking of accessory proteins, is responsible for the extremely fast kinetics of synaptic release events as well as the abnormally high Q_10_ value of ~6 which is most readily explained by a large entropic barrier to fusion^43^.

## Methods

### SV preparation

SVs were prepared from frozen calf brain obtained from Elizabeth Locker Plant Inc. as described previously (Arthur and Stowell, 2007). Briefly, 20 g of frozen tissue was homogenized in a buffer solution (4 mM HEPES (pH 7.3), 0.32 M sucrose). Homogenate was centrifuged at 800 *g* for 15 minutes (4 °C). Supernatant was removed and centrifuged at 9,200 *g* for 20 minutes (4 °C). The pellet was resuspended in an equivolume of homogenization buffer and centrifuged at 10,200 *g* for 20 minutes (4 °C). Pellet was resuspended in 40 ml of homogenization buffer. The resuspended pellet was diluted 1:10 with ice cold dH_2_O. This suspension was subjected to three up-and-down strokes in a glass homogenizer. The resulting lysate was poured rapidly into 9 ml of 1 M HEPES (pH7.3), and the suspension was incubated on ice for 30 minutes. The suspension was then centrifuged at 25,000 *g* for 20 minutes (4 °C), the supernatant was removed and centrifuged at 165,000 *g* for 2 hours (4 °C). The pellet was resuspended in 10 ml of 40 mM sucrose and the suspension was layered on top of a continuous sucrose gradient. Sucrose gradient was centrifuged at 65,000 *g* for 5 hours (4 °C). At the end of centrifugation the gradient revealed a broad band of high turbidity at the 200–400 mM sucrose region, which, from previous experiments^44^ is known to be enriched in SVs. This band was collected and further processed for SYP/VAMP2 complex purification.

### SYP/VAMP2 purification

Purified SVs were incubated for 30 minutes at 0 °C (at 5 mg/ml) in a solution containing 5 mM NaH_2_PO_4_ (pH6.8), 0.2% Triton X-100 (w/v) and 1% cholesterol. The Triton extract was centrifuged at 45,000 *g* for 30 minutes at 4 °C. Triton supernatant was applied to a dry hydroxyapatite/celite column (2:1 w/w) (0.1 g/mg protein) and eluted with solubilization buffer. The resin bound proteins were eluted and found to contain no SYP. The SYP/VAMP2 containing flow through was applied to a POROS H10 anion-exchange column and eluted using a NaCl gradient from 0–1 M (20 mM NaH_2_PO_4_ (pH6.8), 0.2% Brij-35, 0.1% Cholesterol, 40 mM sucrose). 1 ml fractions were collected and analyzed using SDS-PAGE. Fractions containing SYP/VAMP2 were pooled and applied to a Hi-Prep Sephacryl S-300 size-exclusion column calibrated with BIORAD gel filtration standards covering 1,350 to 670,000 daltons. Protein was eluted using 20 mM NaH_2_PO_4_ (pH6.8), 0.2% Brij-35, 0.1% Cholesterol, 40 mM sucrose. Fractions were analyzed using SDS-PAGE and western blotting. Blots were probed with monoclonal SYP and VAMP2 antibodies from Synaptic Systems and fractions of purified SYP/VAMP2 were combined and utilized for EM analysis following SDS-PAGE and western blot analysis. The SYP/VAMP2 complex eluted with an apparent molecular weight of ~400 kD.

### Densitometry

Relative protein quantifications were performed with the gel analysis tool packaged with ImageJ. Measurements were taken in triplicate, averaged and normalized to SYP and VAMP2 standards with concentrations determined using a BCA assay^45^.

### Alignments

Human sequences of SYP and homologs were obtained from the Uniprot database (uniprot.org). The Paralog tree was produced with the simple analysis tool from phylogeny.fr. ClustalW alignment was performed with gap penalties 12 open and 1 extension and aligned with PSI-BLAST at 3 iterations and an E-value cutoff of 0.01. This alignment was assigned a similarity score at each position by the PRALINE server using the BLOSUM62 matrix. The conservation scores were presented at each position as a moving average over a 3 residue window to smooth the plot. The query sequence (hSYP) was then analyzed for hydropathy using the ExPASy ProtScale tool with the Kyte & Doolittle algorithm at a window size of 19 residues.

### Electron microscopy

Imaging was performed as described previously^13^. The final model was a result of 8 rounds of refinement and contained 1232 particles. The resolution of the final model was calculated to be ~28 Å based on FSC using a standard cutoff of 0.5. The absolute hand of the structure was not determined experimentally but was placed in the same hand as the previous SYP structure^13^. Purified SYP/VAMP2 (50 μg/ml) was applied to a freshly carbon coated, glow-discharged EM grid and stained using 2% ammonium molybdate^46^. Images were recorded under low-dose conditions at a magnification of 50,000 and at a defocus range of 1–3 μm using a Tecnai F20 microscope at 200 keV equipped with a Gatan 2 K × 2 K CCD camera. The pixel size was 0.45 nm. Maps were displayed at 2σ resulting in a molecular volume of 226,000 Å^3^ for SYP and 290,000 Å^3^ for the SYP/VAMP2 complex. Difference maps were calculated in CHIMERA following interpolation of the SYP map to the SYP/VAMP2 map resolution and then rotational and translation alignment of density above the 2σ cutoff. The positive difference map was calculated between all map voxels above the 2σ cutoff and displayed according to the approximate calculated molecular volume of the VAMP2 dimer transmembrane helices.

### Image processing

Individual SYP/VAMP2 particles were selected and boxed, and image analysis was performed using the EMAN2 image processing package^47^. Briefly, an initial model was generated using the raw boxed particles and an imposed six-fold symmetry. From this initial model angular projections were generated and particles were again classified based on these projections. From these classifications a new model was generated and the process was repeated. The final model was a result of 10 rounds of refinement and contained 1437 particles. The resolution of the final model was calculated to be ~28 Å based on FSC using a standard cutoff of 0.5.

### Structural alignment and density fitting

The SYP sequence was aligned with the connexin26 sequence based upon a hydropathy window using AlignMe. The aligned sequences were then used in Medeller (http://opig.stats.ox.ac.uk/webapps/medeller/) either as the core SYP 21–220 or the full length sequence as the target and connexin-26 (PDB 2ZW3) as the template. The new model was then dynamically fit to the map using the AD-ENM fitting routine (http://enm.lobos.nih.gov/). This subsequent Cα model was then run through the rotamer search program MaxSprout (http://www.ebi.ac.uk/Tools/structure/maxsprout/) to place side chains. Finally, the entire model was minimized using the 3D refine server (http://sysbio.rnet.missouri.edu/3Drefine/).

## Additional Information

**How to cite this article**: Adams, D. J. *et al.* Architecture of the Synaptophysin/Synaptobrevin Complex: Structural Evidence for an Entropic Clustering Function at the Synapse. *Sci. Rep.* **5**, 13659; doi: 10.1038/srep13659 (2015).

## Figures and Tables

**Figure 1:**
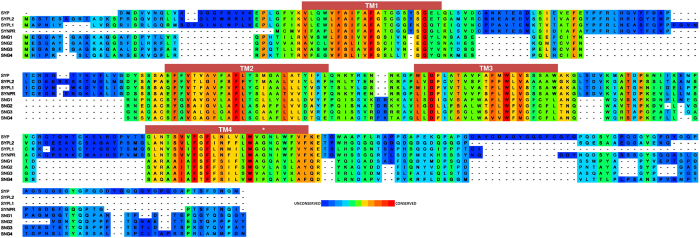
SYP family protein sequence conservation. ClustalW alignment of human SYP and paralogs colored by heat map conservation at each position assigned by the PRALINE server using the BLOSUM62 similarity matrix. The predicted TM helices based on hydropathy are shown above the sequence. G217 of SYP is located in TM4 and marked (*).
